# An Operation on the Lower Jaw

**Published:** 1870-08

**Authors:** C. F. Maunder

**Affiliations:** Surgeon to the London Hospital.


### ARTICLE VIII.

### *An Operation on the Lower Jaw.*

By C. F. Maunder,
Surgeon to the London Hospital.

From various causes, more or less of the lower jaw may demand removal, and one great aim on the part of the Surgeon should be to disfigure his patient as little as possible. Tumours of various kinds call for operative interference, and, if growing from the alveolus, can be easily cut away, leaving the line of the base of the jaw intact, and without any external wound of the cheek. So far as I am aware, it has been customary to incise the cheek more or less with a view to the removal of half the base of the jaw, for example, or for the removal of the ramus, so large an extent of jaw, as half the base not having been hitherto taken away through the mouth. In speaking of these operations on the jaw, I make a distinction between ablations of portions of jaw for tumour and the mere extraction of a sequestrum. In the former instance a careful dissection is necessary in order to separate the healthy attachments of muscles, while, in the latter case (necrosis), the bone is lying more or less loose, and requiring only a certain amount of pulling and twisting to get it out of its bed. I have assisted at the removal of the *whole* lower jaw, become necrosed in the person of a lucifer-match maker. The dead bone was cut in two at the symphysis, without section of integument, and then either half, having been seized with forceps, was twisted out of its place, and brought out through the mouth,

With a view to prevent deformity, and with a hope that some new bone may be developed, I have recently (March, 1870) removed a portion of the lower jaw comprised between the line of the right canine tooth and the middle of the left ramus, without wounding the cheek at all, and preserving a good share of the periosteum. The patient is a female 10 years of age, and the left base of the jaw was enlarged to the size of a hen's egg, the swelling projecting both outwards and inwards to the floor of the mouth, displacing the tongue somewhat, and deforming the lower part of the face. The tumour had a history of two years, had enlarged a good deal of late, was painless, smooth on the surface, and hard and resistant to the touch. It gave one the impression that a growth within was expanding the jaw. I did not pretend to make a diagnosis beyond suggesting that it was probably benign, and that the first part of my operative procedure would be exploratory. I thought it possible that I might have to deal either with a cyst or with a fibrous or cartilaginous growth, and, if so, that I might be able to evacuate the one and enucleate the other, and at the same time preserve the line of the jaw unbroken; but in this I was disappointed.

*Operation.*–Chloroform having been administered, I drilled a hole into the tumour through the mucous membrane covering it, and proved it to be a soft solid within hard walls. I then broke down a portion of this wall with chisel and mallet, and having extracted a portion of the growth, handed it over to Messrs. Sutton and Tay for immediate examination with the microscope. They reported the growth to be myeloid in its nature, and I then proceeded to isolate the portion of bone involved in the disease. The integuments of the chin, including muscular attachments and periosteum, were first turned down off the symphysis, partly with a scalpel and partly with a raspatory, so as to expose the right side of the jaw opposite the right canine tooth (which was then extracted by Mr. A. W. Barret) to the action of the saw and cutting forceps. The soft parts were protected from injury by a spatula passed up through the floor of the mouth behind the bone. The section here being completed, the gum and mucous membrane were severed down to the bone, and a raspatory introduced separated the periosteum, and with it muscular attachments to the required extent, and now, the lower half of the left ramus being bared, this process of bone was cut across with forceps. While affecting this latter step, and using the bone as a lever to aid the application of the forceps, the base broke in two, being now a mere shell of bone. The ramus also was splintered slightly by the forceps. One or two touches with the knife, and the now loosened bone was detached completely. Bleeding was trifling, no ligature being requisite. The muscles of the tongue had been severed at the back of the symphysis, and, to secure this organ forwards, the ligature, put through the tip for a similar purpose during the operation, was fastened to a hare-lip pin passed through the integument of the chin.

For several days subsequent to the operation the mouth was repeatedly syringed with Condy's fluid and water, and fluid nourishment was plentifully administered. The case has progressed favorbly throughout, and, although three weeks only have elapsed since the operation, the patient can protrude the tongue, can articulate tolerably well, and has been running about during some days past.—*London Medical Times & Gazette.*

